# Artificial intelligence in pediatric behavioral health

**DOI:** 10.1186/s13034-023-00586-y

**Published:** 2023-03-12

**Authors:** Gerrit van Schalkwyk

**Affiliations:** Intermountain Health, Salt Lake City, 84102 USA

As we continue to face a growing need for mental health services for children and adolescents, innovative solutions are needed to improve the accessibility and quality of care. One promising solution is the use of technology and artificial intelligence in mental health care. One such tool is ChatGPT, a language model trained by OpenAI that can provide evidence-based information and guidance on a wide range of mental health topics.

ChatGPT has the potential to support pediatric behavioral health in a number of ways. First, ChatGPT can be used as a resource for mental health professionals who are seeking guidance on evidence-based treatments, assessment tools, and best practices for treating common pediatric behavioral health conditions such as anxiety, depression, and attention-deficit/hyperactivity disorder (ADHD). Mental health professionals can use ChatGPT to access information and resources quickly and easily, which can improve their ability to provide effective care to children and adolescents.

Second, ChatGPT can be used as a tool to increase access to mental health services for children and adolescents. Many children and adolescents who are in need of mental health services do not receive them due to barriers such as stigma, lack of access to care, or inadequate mental health care infrastructure. ChatGPT can provide children and adolescents with a non-judgmental and accessible resource for mental health information and guidance. Children and adolescents can use ChatGPT to learn about mental health conditions, coping strategies, and treatment options, which can help them better understand and manage their mental health.

Third, ChatGPT can be used to provide parents and caregivers with guidance and support in managing their child’s mental health. Parents and caregivers play a critical role in supporting their child’s mental health, but they may lack the knowledge and resources to do so effectively. ChatGPT can provide parents and caregivers with evidence-based information on topics such as how to talk to their child about mental health, how to support their child’s treatment, and how to promote positive mental health behaviors in their child.

While ChatGPT has the potential to support pediatric behavioral health in a number of ways, there are also potential limitations to the use of this technology. One limitation is that ChatGPT is not a substitute for a trained mental health professional. While ChatGPT can provide evidence-based information and guidance, it cannot provide a diagnosis or treatment plan tailored to an individual's specific needs. Therefore, it is important for individuals to seek the advice of a mental health professional for a comprehensive evaluation and treatment plan.

Another limitation is that ChatGPT may not be able to address all the complex factors that can impact a child’s mental health. For example, ChatGPT may not be able to address issues related to socioeconomic status, family dynamics, or cultural factors that can impact a child’s mental health. Therefore, it is important to use ChatGPT as a tool in conjunction with other resources and to take a holistic approach to treating pediatric behavioral health conditions.

In conclusion, ChatGPT has the potential to support pediatric behavioral health in a number of ways. By providing mental health professionals, children and adolescents, and parents and caregivers with evidence-based information and guidance, ChatGPT can improve the accessibility and quality of care for pediatric behavioral health conditions. We encourage mental health professionals, parents, and caregivers to explore the use of ChatGPT as a tool for improving pediatric behavioral health.

